# Transforming Grain‐Boundary Brittle Precipitates to Ductility Pathways in Complex Concentrated Alloy

**DOI:** 10.1002/advs.202518465

**Published:** 2026-02-10

**Authors:** Zhixin Li, Xiao‐Tong Li, Zhaoqi Chen, Yushan Geng, Hao Gong, Sijia Hu, Wenli Song, Wanshun Xia, Chuanzheng Li, Linfa Peng, Yue Fan, Pengfei Guan, Bo Zhang, Weihua Wang, Yong Yang

**Affiliations:** ^1^ Department of Mechanical Engineering City University of Hong Kong Hong Kong P. R. China; ^2^ Songshan Lake Materials Laboratory Dongguan Guangdong P. R. China; ^3^ Advanced Interdisciplinary Science Research (AiR) Center Ningbo Institute of Materials Technology and Engineering Chinese Academy of Sciences Ningbo P. R. China; ^4^ State Key Laboratory of Advanced Marine Materials Ningbo Institute of Materials Technology and Engineering Chinese Academy of Sciences Ningbo P. R. China; ^5^ Dongguan Institute of Materials Science and Technology Chinese Academy of Sciences Dongguan China; ^6^ State Key Laboratory of Solid Lubrication Lanzhou Institute of Chemical Physics Chinese Academy of Sciences Lanzhou P. R. China; ^7^ Institute of High Energy Physics Chinese Academy of Science (CAS) Beijing P. R. China; ^8^ Spallation Neutron Source Science Center Dongguan Guangdong P. R. China; ^9^ Institute of Superalloys Science and Technology School of Materials Science and Engineering Zhejiang University Hangzhou Zhejiang P. R. China; ^10^ State Key Laboratory of Mechanical System and Vibration Shanghai Jiao Tong University Shanghai P. R. China; ^11^ Department of Mechanical Engineering University of Michigan Ann Arbor Michigan USA; ^12^ Beijing Computational Science Research Center Beijing P. R. China; ^13^ Department of Materials Science and Engineering City University of Hong Kong Hong Kong P. R. China

**Keywords:** complex concentrated alloys, grain boundary engineering, interfacial plasticity

## Abstract

Conventional wisdom holds that hard grain‐boundary (GB) precipitates embrittle structural alloys by acting as crack initiation sites. In this work, we overturn this paradigm through atomic‐scale interfacial engineering, transforming brittle GB phases into ductility pathways in a machine‐learning identified model complex concentrated alloy. By precisely tailoring thermomechanical processing, we fabricated compositionally and structurally graded interfaces (GIs) that enable sequential plasticity activation and coordinated deformation across GBs. This interfacial architecture converts an intrinsically brittle multi‐phase alloy into a ductile material, achieving an exceptional yield strength of ∼1.2 GPa with a total elongation of ∼20%. The achieved strength‐ductility synergy, realized via interfacial plasticity programming, establishes a generalizable materials design strategy to overcome the persistent challenge of GB embrittlement in precipitation‐strengthened alloys.

## Graphical abstract

By engineering graded BCC/L1_2_ interfaces, brittle precipitates in a complex concentrated alloy enable sequential deformation, realizing gigapascal strength with >20% elongation to solve the strength‐ductility trade‐off.

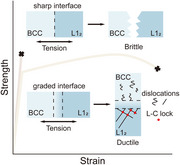

## 1 Introduction

Complex concentrated alloys (CCAs) have revolutionized structural materials design by transcending traditional property trade‐offs, achieving unprecedented combinations of strength, ductility, and high temperature resistance [1, 2, 3, 4]. Severe lattice distortion and sluggish diffusion, though not universal to all CCAs, are common features in many systems that, when present, effectively facilitate the dynamic regulation of phase stability and unique deformation pathways [5, 6, 7]. Yet, the formation of brittle secondary phases (e.g., intermetallics, carbides, or topologically close‐packed phases) during alloy fabrication or service remains a critical barrier to their widespread applications [8, 9]. While such precipitates can enhance strength, their role as stress concentrators accelerates crack nucleation and propagation, catastrophically degrading ductility and fracture resistance [10]. In conventional alloys, brittle phases are mitigated through compositional fine‐tuning or thermomechanical processing, guided by their accurately predicted phase diagrams [11, 12]. However, CCAs’ intrinsic chemical complexity disrupts non‐equilibrium phase predictions due to trapped dynamics [13], making brittle precipitate suppression or modification exceptionally challenging. Unexpected brittle phases induce catastrophic embrittlement, particularly at GBs where they disrupt dislocation dynamics and interfacial cohesion [9, 14, 15, 16, 17, 18]. Current strategies to address this issue focus on two fronts: (1) preventing brittle phase formation via alloy design (e.g., adjusting overall valence electron concentration) [19, 20] and (2) modifying precipitate characteristics (e.g., size, morphology, distribution, and interfacial structure) through thermomechanical process optimization (e.g., rapid solidification, aging treatments) [21, 22, 23, 24]. Notably, interfacial engineering, such as tailoring grain boundary chemistry or introducing coherent/semi‐coherent interfaces between precipitates and the matrix, has shown promise in alleviating the detrimental effects of brittle phases [10, 25, 26, 27, 28].

Here, through atomic‐scale interfacial engineering, we introduce a method that can transform brittle grain boundary precipitates into ductility pathways in a model complex concentrated alloy. Specifically, by creating GIs between disordered body‐centered cubic (BCC) and ordered face‐centered cubic (L1_2_) phases, both of which are common in CCAs, we fundamentally alter dislocation interaction mechanisms at these critical junctions. Contrary to classical theories where interfaces impede dislocation motion [29], our gradient‐ordered interface enables cooperative deformation between soft and hard phases, activating unprecedented ductilization. This mechanism simultaneously achieves gigapascal‐level strength and exceptional ductility, previously deemed mutually exclusive in conventional alloys. Our findings overturn the conventional view of interfacial brittle precipitates as embrittlement sources, demonstrating that GIs can transform brittle precipitates into ductility‐enhancing features. By establishing a systematic framework for interfacial engineering in CCAs, this work opens avenues to design next‐generation structural materials that defy the traditional strength‐ductility trade‐off.

## 2 Results

### 2.1 Graded Interface Structure

Building on our machine learning framework for spinodal alloy discovery [30], we synthesized an expensive‐element‐free spinodal alloy with the chemical composition of Ni_59_Cr_24_Al_12_Fe_5_ (at%) through arc melting and homogenization at 1373 K, which is about 84% of the alloy's melting temperature (see Materials and Methods and Figure S1). The homogenized alloy developed an equiaxed microstructure with an average size of 205 ± 132 µm (Figure S2A,B), containing spinodal nanostructures with coherent disordered and ordered face‐centered cubic (i.e., FCC/L1_2_) interfaces (Figure S2C,D). Elemental mapping revealed Cr enrichment in the FCC phase (Figure S2E), consistent with spinodal decomposition thermodynamics. Furthermore, thermodynamic calculations through CALPHAD revealed a stable BCC phase in our alloy below approximately 1200 K (see Materials and Methods and Figure S3), which motivated thermomechanical treatments of cold rolling followed by annealing at targeted temperatures (i.e., 973 and 1173 K) for various time durations (see Materials and Methods). This approach successfully precipitated BCC phases in our alloy.

**FIGURE 1:**
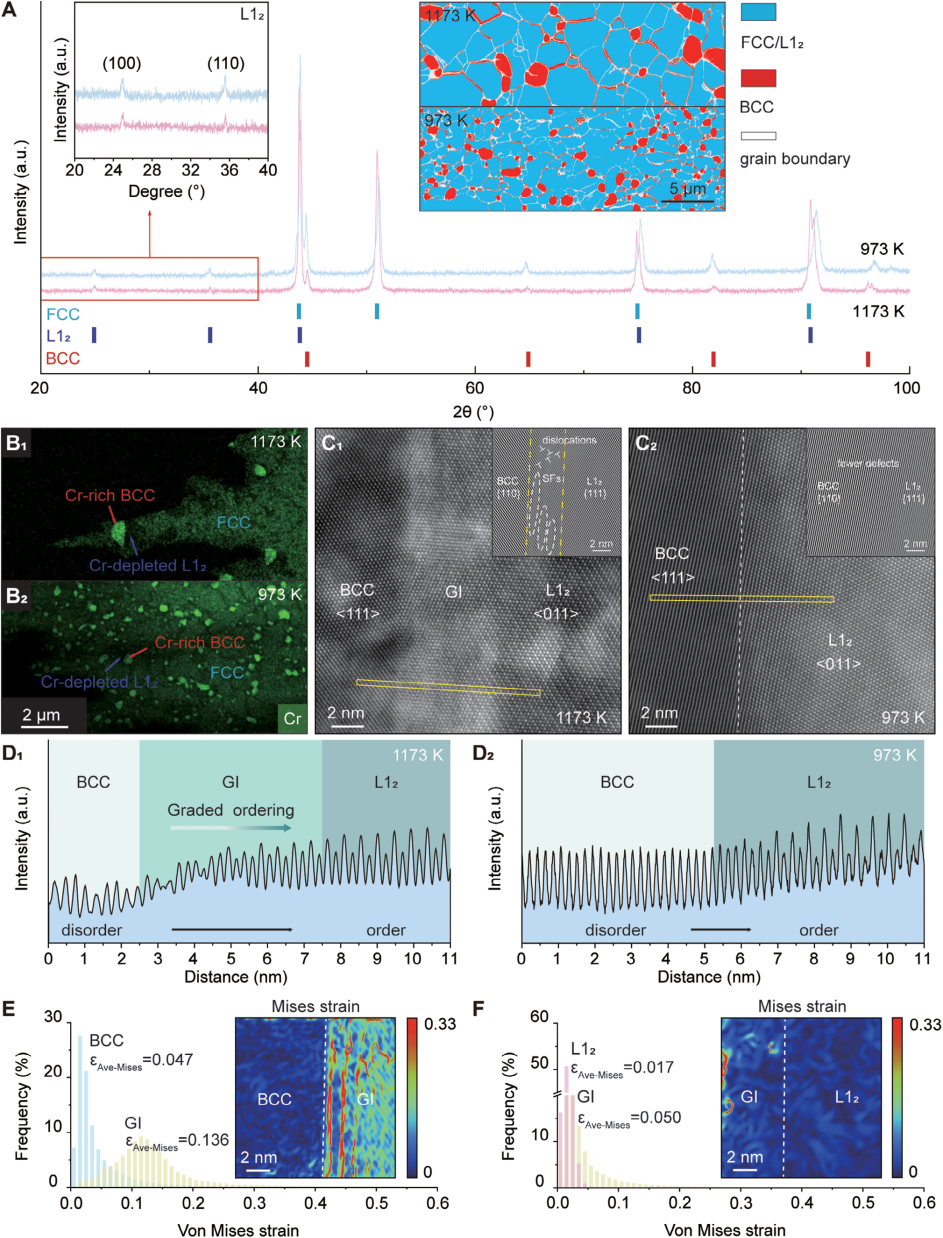
Structural characterization of our machine learning identified alloy annealed at 1173 and 973 K respectively for 8 h. (A) XRD patterns of 1173 K‐ and 973 K‐annealed alloys. The insets show an enlarged view of the region indicated by the red square and EBSD phase maps of 1173 K‐ and 973 K‐annealed alloys. a.u., arbitrary units. (B_1_, B_2_) STEM‐EDS mappings of chromium in ion‐milled 1173 K‐ and 973 K‐annealed alloys. (C_1_, C_2_) Atomic aberration‐corrected HAADF‐STEM images of the interface between BCC and L1_2_ of 1173 K‐ and 973 K‐annealed alloys, respectively, viewed along the zone axis [011] L1_2_. The insets show corresponding IFFT images. (D_1_, D_2_) HAADF line‐scan intensity profile of 1173 K‐ and 973 K‐annealed alloys in the yellow square in (C_1_, C_2_), respectively. (E) Von Mises strain distribution between BCC and GI referred to *g*‐vectors of BCC. The inset shows the corresponding von Mises strain map. (F) Von Mises strain distribution between L1_2_ and GI obtained by referring to *g*‐vectors of L1_2_. The inset shows the corresponding von Mises strain map.

X‐ray diffraction (XRD) analysis confirmed the co‐existence of FCC, L1_2_ and BCC phases in both annealed alloys (Figure 1A), with fully recrystallized equiaxed grains and BCC precipitation along GBs revealed by electron backscattered diffraction (EBSD) (Figure 1A, inset). Additionally, Rietveld refinement of the neutron diffraction patterns was performed to quantify the phase contents (Figure S4): the 973 K‐annealed sample contained 74.9 vol% FCC, 15.4 vol% L1_2_, and 9.7 vol% BCC, while the 1173 K‐annealed sample exhibited 76.2 vol% FCC, 13.9 vol% L1_2_, and 9.9 vol% BCC. Compositional analysis revealed these BCC precipitates to be Cr‐rich (Table S1), forming adjacent to Cr‐depleted L1_2_ phases (Figure 1B_1_,B_2_). This observation, supported by our thermodynamic calculations (see Supporting Text), suggests that Cr segregation (strong negative segregation enthalpy, −20 kJ/mol) to GBs drives BCC formation, while concomitant Cr depletion from the FCC phase promotes local L1_2_ ordering (Figure S3). According to the literature [31], preferential GB precipitation is enhanced by accelerated diffusion at GBs, which was measured to be four to six orders of magnitude faster than in grain interiors. Furthermore, advanced scanning transmission electron microscopy (STEM) characterization revealed striking differences in interfacial structure between annealing conditions. The 1173 K‐annealed alloy exhibited a diffuse BCC/L1_2_ interface (5 to 12 nm wide) with a gradual ordering transition (Figure 1C_1_,D_1_), while the 973 K‐treated sample showed a sharp interface (< 2 nm) (Figure 1C_2_, D_2_), revealed by high‐angle annular dark‐field scanning transmission electron microscopy (HAADF‐STEM). The line scan was acquired via HAADF imaging, a technique leveraging Z‐contrast, where signal intensity is proportional to the square of the atomic number (Z^2^). As such, the resulting profile captures the integrated signal from all constituent elements, enabling direct visualization of atomic ordering and compositional variations across the interface. These observations were corroborated by scanning transmission electron microscopy‐energy dispersive X‐ray spectroscopy (STEM‐EDS) compositional profiles (Figure S5). The TEM tilting experiment confirmed that the observed GI phase represents a genuine structure, rather than an artifact arising from the overlap of two distinct phases (Figure S6). Under edge‐on conditions, the GI phase displays distinct coexisting L1_2_ and BCC diffraction spots, domain width of ∼8 nm. By contrast, HRTEM images acquired at other tilt angles reveal substantial Moiré fringes with widths ranging from ∼15 to 23 nm, which is a clear artifact induced by the inclined overlap of the L1_2_ and BCC phases. Atomic‐scale strain fields were characterized via a semi‐qualitative and semi‐quantitative geometric phase analysis (GPA) (see Materials and Methods, Figure S7), with strain quantification performed using two independent reference lattices (L1_2_ phase and BCC phase) to mitigate biases from single‐reference selection. While the absolute quantitative strain values are reference‐dependent, the strain distribution trend is reliably consistent across both reference frameworks: significant lattice distortion is localized toward the BCC side of the interfaces (Figure 1E,F), and the GI region exhibits higher strain than either pure BCC or L1_2_ phases. This strain feature is accompanied by misfit dislocations and stacking faults (Figure 1C_1_, inset) in the 1173 K‐annealed alloy, whereas fewer defects were observed at the phase interfaces in the 973 K‐annealed sample (Figure 1C_2_, inset). The corresponding inverse fast Fourier transformation (IFFT) images were derived from fast Fourier transformation (FFT) spots indicated by yellow arrows (Figure S8B,D).

### 2.2 Mechanical Properties

**FIGURE 2:**
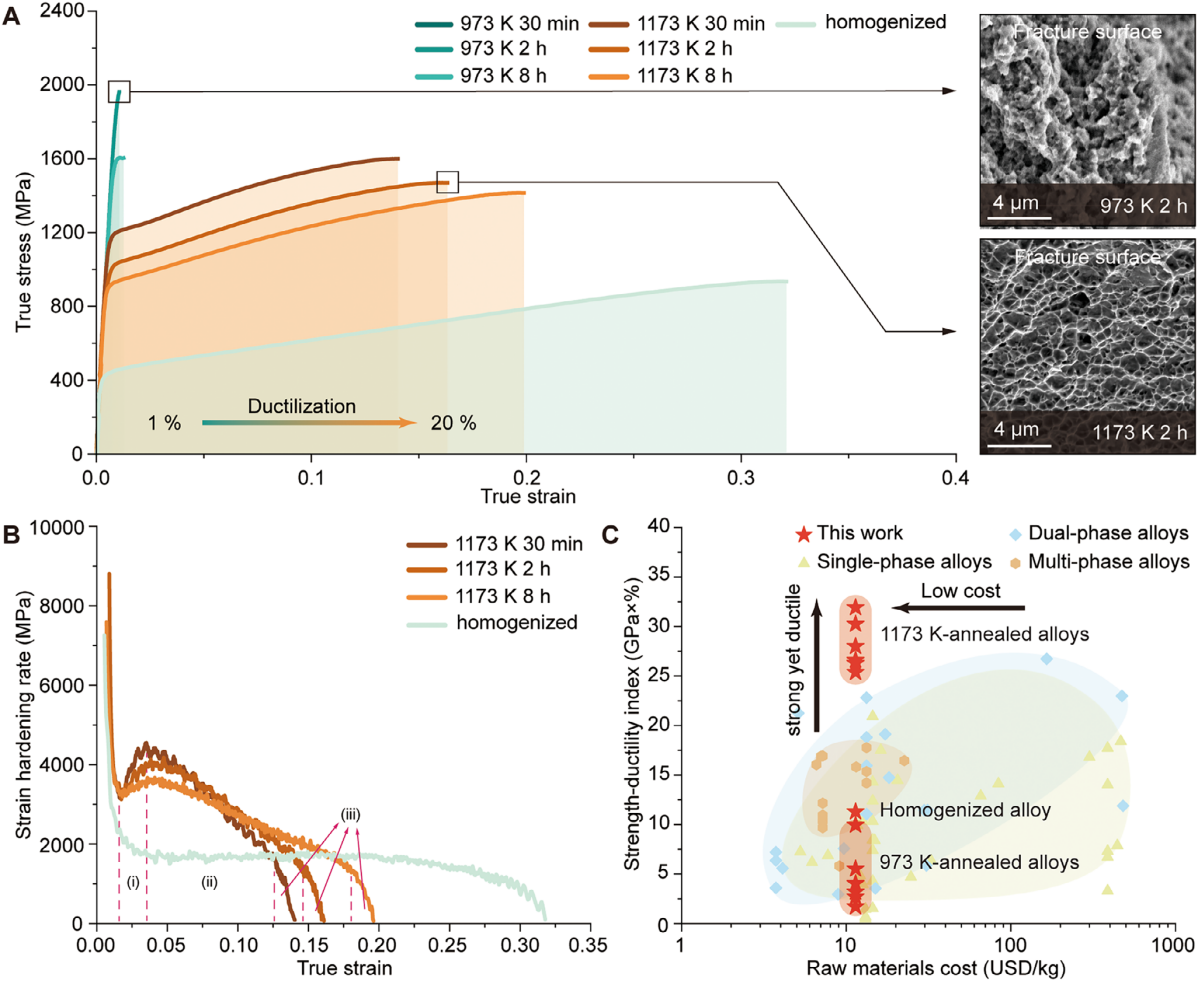
Room‐temperature tensile properties and cost of this alloy and other alloys. (A) Room‐temperature true tensile curve of 1173 K‐annealed alloys, 973 K‐annealed alloys and homogenized alloy. (B) Strain hardening rate versus true strain of 1173 K‐annealed alloys and homogenized alloy. (C) Strength‐ductility index versus raw materials cost of 1173 K‐annealed alloys, 973 K‐annealed alloys and homogenized alloy in this work, compared with other alloys. Unit: United States dollar (USD)/kg. Source data are provided in the supporting information.

Room‐temperature uniaxial tensile tests at a strain rate of 8 × 10^−4^ s^−1^ (see Materials and Methods) revealed a marked increase in yield strength for alloys annealed at 1173 and 973 K compared to the homogenized baseline (Figure 2A). However, the 973 K‐annealed alloys displayed severe embrittlement, attributed to continuous BCC precipitation along GBs. In contrast, the 1173 K‐annealed alloys retained substantial tensile ductility (1% to 20%) despite similar GB precipitation (Figure 2A). Fractographic analysis highlighted this dichotomy: the 973 K‐annealed alloy showed localized fibrous deformation features, while the 1173 K‐annealed counterpart displayed extensive dimple formation (Figure 2A, inset). The strain hardening responses further distinguished the 1173K‐annealed alloys from the homogenized ones (Figure 2B). The 1173 K‐annealed variant exhibited a three‐stage work hardening profile: the first stage is a rising segment from yielding to ∼3% true strain, with a distinct hump (local peak in hardening rate) appearing within this range; this is followed by a gradual decline in hardening rate until the onset of necking, and finally an accelerated decay in the late deformation stage. By contrast, the homogenized alloys exhibit only a conventional two‐stage hardening mode with significantly lower hardening rates. When benchmarked against single‐phase, dual‐phase, and multi‐phase alloys using the strength‐ductility index (e.g., ultimate tensile strength × ductility) [22] and raw materials cost (Supporting Text and Table S2), the 1173 K‐annealed alloys outperform the previously reported alloys, including those containing expensive elements, by achieving both a superior strength‐ductility synergy and lower raw material cost (note: prices reflect temporary market data and are subject to temporal fluctuations) (Figure 2C), which underscores their exceptional balance of mechanical properties and economic viability.

### 2.3 Microstructure Evolution Upon In Situ Tension

**FIGURE 3:**
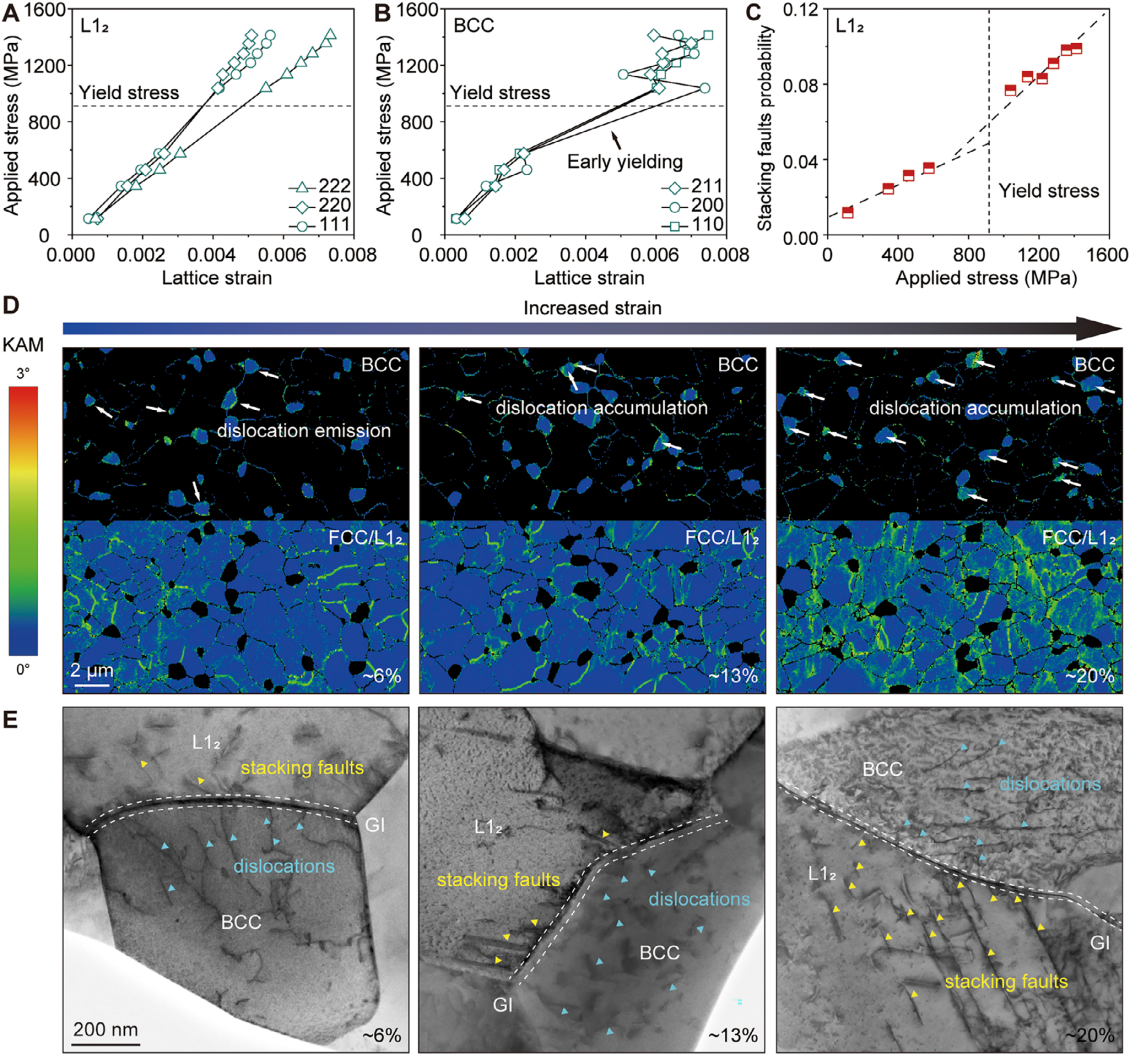
Microstructural evolution of room‐temperature deformed alloy annealed at 1173 K for 8 h. (A, B) Evolution of lattice strain of L1_2_ and BCC, respectively, upon in situ neutron diffraction tension. (C) Stacking faults probability of L1_2_ upon in situ neutron diffraction tension. (D) KAM distribution of BCC and FCC/L1_2_ with increased strain. (E) HAADF‐STEM images with increased strain. Stacking faults are marked with yellow triangles, and dislocations are marked with cyan triangles.

To elucidate the mechanistic origin of enhanced ductility, we investigated the deformation dynamics in both the 1173 K‐ and 973 K‐annealed alloys using in situ neutron diffraction tensile testing at room temperature (see Materials and Methods). In the ductile 1173 K‐annealed alloy, phase‐specific lattice strain analysis revealed that the BCC phase yielded earlier than the L1_2_ phase (Figure 3A). In contrast, despite the overall brittle fracture, the 973 K‐annealed alloy exhibited initial yielding in the L1_2_ phase (Figure S9). As plasticity progressed in the 1173 K‐annealed alloy, the increasing lattice strain difference between the L1_2_ (222) and (111) planes suggested a propensity for stacking fault formation [32, 33]. Quantification of stacking fault (SF) probability (see Supporting Text) revealed a marked increase upon the onset of macroscopic plasticity (Figure 3C). Kernel average misorientation (KAM) mappings illustrated strain distribution across BCC and FCC/L1_2_ phases (Figure 3D). At a strain of 0.06, elevated KAM values localized primarily within BCC phases near interfaces, indicative of dislocation activities in BCC domains [34, 35]. With further straining, dislocation accumulation within the BCC phase was reflected in rising KAM values, while high‐KAM regions expanded around the BCC phase, suggesting coordinated BCC‐L1_2_ deformation. Ex‐situ TEM analysis of specimens deformed to selected strains further revealed asymmetric plasticity initiation mediated by GIs (Figure 3E). At early plasticity (ε = 0.06), dislocations were observed in the BCC phase, accompanied by short SFs in adjacent L1_2_ regions. Upon further deformation, BCC phases developed dense dislocation tangles, whereas L1_2_ phases exhibited extensive stacking fault proliferation.

### 2.4 Molecular Dynamics Simulation

**FIGURE 4:**
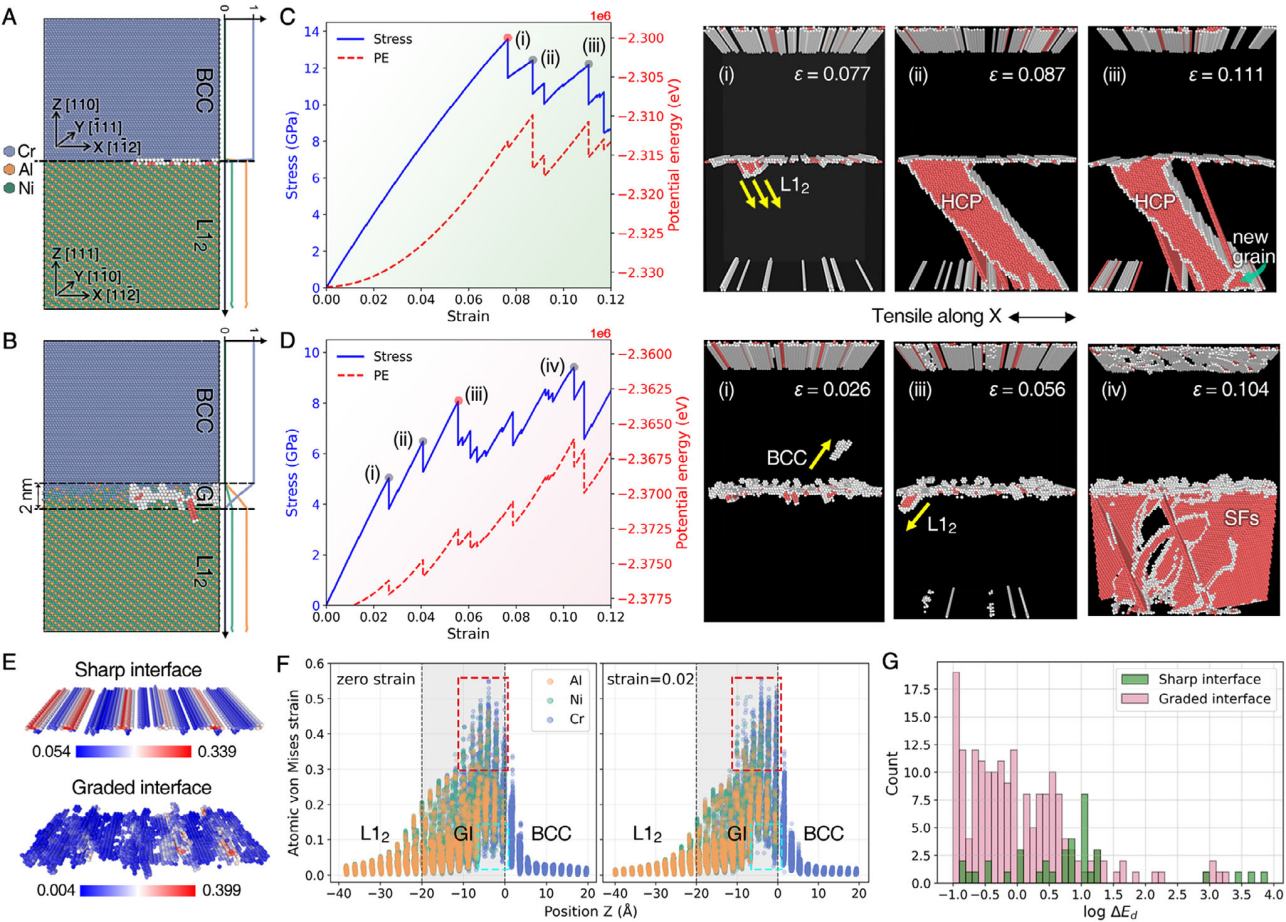
Role of concentration gradient at the BCC/L1_2_ phase interface in mechanical responses. (A, B) Simulation cells featuring the sharp phase interface with the K‐S orientation relationship and the compositionally GI. (C, D) Strain–stress curve and system potential energy evolution under athermal uniaxial tensile loading parallel to the phase interface (along the x‐direction). Corresponding atomic configurations are displayed alongside, with perfect lattice atoms omitted for clarity. (E) Comparison of atomic‐level von Mises strain maps prior to the incipient plasticity. (F) Atomic strain and elemental partitioning near the GI relative to sharp interface before and after loading. (G) Logarithmic histogram of energy dissipation at the two interfaces during deformation.

To further investigate the role of interface structure on deformation mechanisms, we employed molecular dynamics (MD) simulations comparing mechanical responses across sharp and compositionally graded BCC/L1_2_ interfaces. Two idealized models were constructed: (1) a sharp interface between Cr (BCC) and Ni_3_Al (L1_2_) phases following the K‐S orientation relationship (Figure 4A), and (2) a GI maintaining the K‐S relationship while incorporating a 2 nm Cr‐concentration gradient on the L1_2_ side (Figure 4B, Materials and Methods). Energy minimization revealed distinct interface structures: the sharp interface developed a dislocation array, while the GI accommodated complex defects including point defects, dislocations, and stacking faults (SFs), which were consistent with our experimental observations (Figure 1C_1_,C_2_). Under uniaxial tension along the x‐direction (periodic boundary conditions), the GI demonstrated markedly different mechanical behaviors compared to the sharp interface, as evidenced by their stress‐strain responses and corresponding atomic configurations (Figure 4C,D).

The sharp interface model (Figure 4C) exhibits global yielding at the strain of 0.077, initiated by simultaneous nucleation and rapid propagation of multiple dislocations within the L1_2_ phase owing to its intrinsically lower unstable stacking fault energy (Figure S10). These dislocations become arrested at adjacent interfaces, generating HCP lamellae. At higher strains (strain > 0.1), sustained dislocation emission from interfaces leads to the complete transformation of the L1_2_ phase into the HCP structure. In contrast, the GI (Figure 4D) demonstrates fundamentally different behavior. Plasticity initiates earlier at a strain of 0.026 through initial stress relaxation from the BCC‐phase dislocation emission, followed by secondary stress relaxation from dislocation nucleation at alternate interface sites. Remarkably, this BCC‐dominated plasticity coexists with continued strain hardening. Global yielding occurs at the strain of 0.056 when dislocations propagate into the L1_2_ phase. Subsequent deformation proceeds through coordinated dislocation and SF activities in the L1_2_ phase, where SF propagation and annihilation sustain strain hardening, a mechanism absent in the sharp interface system.

To elucidate the mechanistic contrast between interface types, we analyzed atomic‐scale strain distributions prior to plasticity onset (Figure 4E). The sharp interface exhibits strain localization aligned with its pre‐existing dislocation array, while the GI shows heterogeneous strain dispersion that promotes interface‐mediated plasticity. Quantitative comparison of von Mises strain (Figure 4F) reveals asymmetrical distortion across the GI: the BCC side experiences significantly greater atomic strain than the L1_2_ side in the direction perpendicular to the interface (the z‐direction). This asymmetry was also observed in our experiments (Figure 1E,F) and stems from differential interface effects – while the L1_2_ phase accommodates only chemical order transition, the BCC phase sustains concurrent chemical and structural frustration. Mechanical loading amplifies this asymmetry, as evidenced by strain redistribution (Figure 4F): increased data density (red box) indicates enhanced BCC‐side distortion, while decreased density (cyan box) reflects L1_2_‐side stabilization. This creates a rugged energy landscape on the BCC side that preferentially enables irreversible atomic rearrangements. Consequently, when interfaces are graded, plasticity initiates in the BCC phase rather than the L1_2_ phase. The corresponding plasticity initiation phenomenon can likewise be observed during in situ neutron diffraction (Figure 3A). From the energy perspective, external work is initially stored as elastic potential energy during mechanical deformation of solids, while plastic deformation dissipates energy through non‐affine atomic rearrangements. The dissipated energy per unit strain increment (Δ*E_d_*), calculated as the difference between external work and potential energy variation (see Supporting Text), reveals fundamentally distinct dissipation profiles between the sharp and GI models (Figure 4G). The sharp interface exhibits discrete energy dissipation events, while the GI shows a continuous, multi‐order distribution. Notably, the GI demonstrates significantly higher event density (i.e., log Δ*E_d_* < 1.0, corresponding < 10 eV), indicating abundant low‐energy dissipation sources that promote distributed plasticity. This dense population of small dissipation events also explains the enhanced ductility observed in GI systems.

## 3 Discussion

**FIGURE 5:**
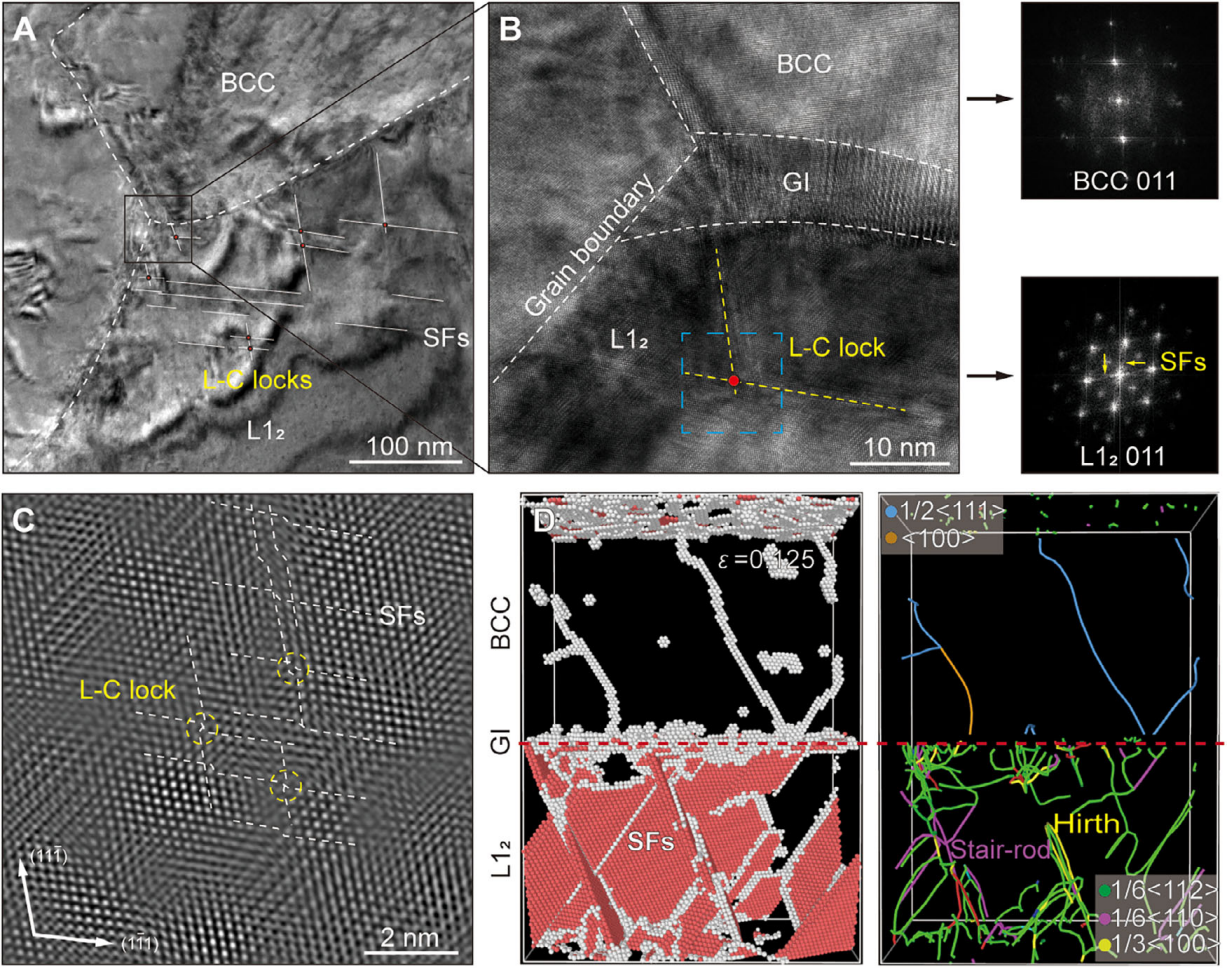
Stacking faults network and Lomer‐Cottrell (L‐C) locks during hardening in alloy annealed at 1173 K for 8 h. (A) Bright‐field TEM image of stacking faults (SFs) and L‐C locks in the deformed L1_2_ phase. (B) HR‐TEM image of black square in (A) with corresponding FFT images. (C) IFFT image of blue dashed square in (B). (D) Simulation snapshots of dislocation activities at a strain level of 0.125, identified using the Dislocation Analysis Algorithm (DXA). Sessile dislocations, such as stair‐rod and Hirth dislocations, formed as a result of SF interactions, acting as topological barriers and contributing to the hardening behavior.

While the BCC precipitates remain almost the same in both 973 K‐ and 1173 K annealed alloys, our findings demonstrate that GIs can fundamentally alter the deformation paradigm in multiphase alloys by transforming the hard and brittle BCC precipitates from fracture initiation sites into active contributors to ductility through sequential plasticity activation. The significant mechanical difference between the two annealed alloys is not caused by grain size variation, as the grain strengthening contributions calculated via the Hall‐Petch equation are ∼800 MPa and ∼1100 MPa for the 973 and 1173 K‐annealed samples, respectively (see Supporting Text). Additionally, no obvious texture is observed in either alloy, with texture intensities quantified as 5.95 and 4.76 (Figure S11), which are weak to induce the remarkable mechanical discrepancy. This interface‐mediated deformation programming enables exceptional strain hardening, exhibited as “hump” feature (Figure 2B), by establishing a spatially and temporally coordinated plasticity sequence across phases (Figure 3A). The “hump” feature arises from the competition between the exceptional strain hardening of GI and the traditional dynamic recovery softening mechanism. The process initiates with preferential dislocation nucleation in BCC domains at GIs (Figure 4D), followed by stress‐dependent slip transmission that activates coordinated faulting in adjacent L1_2_ phases. A critical feature of this mechanism emerges at stacking fault intersections, where topological barriers (e.g., stair‐rod dislocation reactions, Figure 5) temporarily arrest plasticity while efficiently redistribute stress back to BCC domains [36, 37, 38]. This dynamic stress redistribution establishes an oscillatory hardening cycle: when dislocation activity in BCC phases approaches local hardening capacity limits, the resulting stress concentration triggers compensatory plasticity in previously undeformed L1_2_ regions. The process is facilitated by gradient‐induced lattice distortion, which lowers the energy barrier for new fault nucleation in pristine L1_2_ regions, while residual strain accommodation in BCC domains preserves L1_2_ structural integrity for subsequent deformation events, as confirmed by MD simulations (Figure 4D).

This sophisticated deformation mechanism stands in stark contrast to conventional alloy behavior [39, 40, 41, 42] and our control samples lacking GIs, where plasticity remains confined exclusively to L1_2_ phase (Figures 4C; S9H). In these systems, the BCC phase maintains almost purely elastic deformation, ultimately leading to interfacial fracture, as corroborated by both in situ neutron diffraction tensile tests (Figure S12) and TEM observations that reveal dislocation‐scarce BCC regions in fractured samples (Figure S9D). Bulk‐loading simulation on L1_2_ phase further shows a markedly delayed plastic onset at significantly higher global strain levels, with stacking faults nucleating primarily on the same slip plane rather than forming coordinated multi‐plane interactions (Figure S13), underscoring the role of GIs in promoting early plasticity and strain hardening. The GI strategy presented here suggests a general materials design principle for overcoming the strength‐ductility trade‐off in precipitation‐strengthened alloys, demonstrating how traditionally brittle components can be transformed into active participants in deformation processes through interface engineering.

In addition to the exceptional mechanical behavior, the 1173 K‐annealed alloys exhibited remarkable thermal stability at GBs, maintaining an ultra‐fine grain structure with an average size of 1.42 ± 1.37 µm even after prolonged isothermal annealing at 0.72 T_m_ for 120 h. This minimal grain coarsening is significantly lower than that observed in comparable reference alloys (Figure S14). It can be attributed to the synergistic stabilization effect of both BCC‐pinned triple junctions and the constraining influence exerted by the GIs between BCC and L1_2_ phases [43], which collectively immobilized GBs. Following the dissolution of the BCC phase, the alloy undergoes nanoscale spinodal decomposition through atomic diffusion. We propose that achieving nanoscale spinodal decomposition presents greater kinetic challenges than reverting to a solid solution state. Thus, the resulting spinodal structure impedes BCC phase dissolution and thereby indirectly enhances the alloy's thermal stability. Complemented by the suppressed grain coarsening, this exceptional microstructural stability strongly suggests the potential for superior creep resistance compared to conventional alloys. Future work will focus on investigating the alloy's creep properties with tailored GIs to quantitatively assess their role in enhancing high‐temperature performance.

The formation of these distinct GIs stems from a critical synergy between thermodynamic metastability and kinetic acceleration. In our spinodal alloy system, the negative second derivative of Gibbs free energy creates an inherent thermodynamic driving force for spontaneous compositional decomposition [44]. However, the kinetic pathway determining the final microstructure is equally crucial: cold rolling generates a high density of dislocations that preferentially tangle at spinodal phase boundaries and GBs. During annealing, these dislocation networks serve as pipe diffusion highways [45], enabling rapid elemental redistribution despite the relatively low annealing temperature. We further emphasize that the annealing temperature was meticulously optimized to approach — but not exceed — the BCC solvus boundary. This strategic thermal profile achieves two key objectives: (1) maximizing atomic mobility while (2) preserving the BCC phase as a stabilizing scaffold. The combination of dislocation‐accelerated diffusion and precisely controlled thermal activation synergistically broadens the element diffusion width [46, 47], creating the observed nanoscale interface architecture. We believe that such GI structures are fundamentally generalizable to other alloy systems exhibiting two essential characteristics: (i) thermodynamic instability that drives spontaneous elemental segregation, and (ii) tailored kinetic pathways (e.g., our dislocation pipe networks coupled with optimized thermal processing) that enable sufficient compositional gradients.

## 4 Conclusions

This work develops a generalizable atomic‐scale interfacial engineering strategy to resolve the GB embrittlement issue in complex concentrated alloys CCAs. By tailoring thermomechanical processing, we transform brittle Cr‐rich BCC precipitates at GBs into ductility‐enhancing pathways via BCC/L1_2_ GIs, which enable sequential plasticity activation and dynamic stress redistribution across phases. The GI‐engineered alloy achieves an exceptional combination of ∼1.2 GPa yield strength and ∼20% fracture elongation, breaking the strength‐ductility trade‐off in precipitation‐strengthened alloys. It also maintains remarkable thermal stability, retaining an ultra‐fine grain structure after prolonged high‐temperature annealing. The GI formation relies on synergistic thermodynamic metastability and kinetic regulation, which is generalizable to other alloy systems with spontaneous elemental segregation and tailored diffusion pathways. The resulting microstructure delivers an unprecedented combination of properties — simultaneous high strength, enhanced plasticity, and exceptional thermal stability — by design. These advances directly address critical materials challenges in extreme environments, from turbine blades during thermomechanical fatigue in next‐generation jet engines and nuclear reactor components requiring irradiation‐resistant dimensional stability.

## 5 Materials and Methods

### 5.1 Alloy Design

The computational design of alloys targeting spinodal decomposition was performed using an artificial neural network (ANN) model implemented in MATLAB R2024b (The MathWorks, Inc.). The compositional space was constrained to the following atomic percentage ranges: Ni (25–65), Cr (5–35), Al (5–35), and Fe (5–35), with a resolution of 1 at. %. Under these predefined compositional constraints, a database encompassing 23,787 distinct quinary alloy compositions was generated, ensuring each composition summed to 100 at. % and consisted solely of integer percentages. As a critical preprocessing step for thermodynamic stability targeting the FCC + L1_2_ dual‐phase microstructure via spinodal decomposition, compositions were screened based on two key criteria:

(1) Valence electron concentration (VEC), calculated using the formula:
$$VEC=\sum_{i=1}^{4}\left(c_{i}VEC_{i}\right)$$

where *c_i_* is elemental concentration, *VEC_i_* is the VEC for a specific element. Compositions with VEC > 8.0 were retained.

(2) Spinodal decomposition likelihood, predicted by the ANN model we developed [30] to have a high probability (> 0.98) of undergoing spinodal decomposition were retained.

Only compositions satisfying both criteria (VEC > 8.0 and spinodal likelihood > 0.98) were selected for further consideration, as this combination was predicted to thermodynamically favor the formation and stabilization of the desired FCC + L1_2_ spinodal structures.

### 5.2 Sample Preparation

Ni_59_Cr_24_Al_12_Fe_5_ (at. %) ingots were arc‐melted in high‐purity argon atmosphere. To ensure homogeneity, each ingot was re‐melted and flipped at least six times. Raw materials purity exceeded 99.95 wt.%. Prior to melting, the chamber was evacuated to < 3 × 10^−3^ Pa and backfilled with high‐purity argon. Residual oxygen was scavenged by melting a Ti getter at least two minutes. Following multiple remelts, molten alloys were drop‐cast into a water‐cooled copper mold with dimensions of 5 × 12 × 60 mm^3^. All as‐cast sheets were homogenized at 1373 K for 8 h, followed by water quenching. The homogenized sheets were cold‐rolled to reduce the thickness by 80% reduction, followed by annealing at 873–1173 K from 15 min to 120 h. The details of processed samples are provided in Table S2.

### 5.3 Mechanical Testing

Room‐temperature uniaxial tensile properties were evaluated using and Instron mechanical tester (34TM‐30, Instron) at a constant strain rate of 8 × 10^−4^ s^−1^. Dog‐bone shaped tensile specimens were machined via electrical‐discharge machining (EDM) with nominal gauge dimensions of 12.5 mm length × 2 mm width. Prior to testing, gauge sections were polished with 1500‐grit SiC grinding papers to obtain a clean and smooth surface. For digital image correlation (DIC), polished surfaces were coated with white paint followed by application of a black speckle pattern. Tensile tests were recorded using a Lumix S5M2 camera equipped with a Lumix S 70–300 mm lens. DIC analysis was performed using Ncorr V1.2.2 [48], an open‐source MATLAB R2024b (The MathWorks, Inc.) software.

### 5.4 Microstructural Characterization

Prior to characterization, all samples were mechanically polished with silicon carbide papers (from 120‐grits to 1000‐grits) to remove impurities and obtain a smooth surface. Phase identification was performed using an X‐ray diffractometer (Rigaku, MiniFlex600) with Cu Kα radiation (λ = 1.5406 Å). Scans were acquired over a 2θ range of 20–100° with a scan rate of 5°/min. The fracture surface of specimens was characterized using a scanning electron microscope (SEM, Phenom, Thermo Fisher Scientific). Prior to the electron backscatter diffraction (EBSD) test, all samples were polished with 0.05 µm Al_2_O_3_ suspensions, followed by nano SiO_2_ oxide polishing suspensions for final surface refinement. EBSD scanning was carried out on a field emission SEM (Helios 5UC, Thermo Fisher Scientific) equipped with an EBSD detector (Oxford C Swift, Oxford Instruments). All EBSD data was analyzed using AZtecCrystal V2.1. Transmission electron microscopy (TEM) samples were prepared by two methods: (1) Mechanical thinning to ∼50 µm, punching into 3‐mm discs, and argon‐ion milling to electron transparency (PIPS II 695, Gatan); (2) Site‐specific lift‐out using a dual‐beam focused ion beam (FIB, Helios 5UX, Thermo Fisher Scientific) when required. The atomic‐resolution microstructure was analyzed by TEM, (JEM‐F200, JEOL) and aberration‐corrected TEM (Spectra 300, Thermo Fisher Scientific) both equipped with high angle annular dark field (HAADF) detectors and energy‐dispersive X‐ray (EDX) spectrometers.

### 5.5 Thermodynamic Calculations

All phase diagrams and compositional diagrams in this work were calculated using CALPHAD methodology implemented in Thermo‐Calc 2024b software with the TCHEA8 thermodynamic database. This database incorporates optimized Gibbs free energy descriptions for all relevant phases (e.g., FCC, L12, BCC and liquid), with calculations spanning 700–2000 K.

### 5.6 In Situ Neutron Diffraction Tensile Tests

Room‐temperature in situ neutron diffraction uniaxial tensile tests were conducted on the Engineering Materials Diffractometer (EMD) at the China Spallation Neutron Source (CSNS). Dog‐bone shaped samples with a gauge length of 30 mm and width of 3 mm were machined via EDM. The loading axis was adjusted to 45° relative to the incident neutron beam. The EMD uses time‐of ‐flight detection with two ± 90° detector banks, simultaneously recording diffraction patterns with diffraction vectors parallel and perpendicular to the loading direction. The gauge volume was fully immersed in the 6 mm × 6 mm incident neutron beam. A double‐disk chopper enhanced neutron transmission within 0.8–3.8 Å wavelength bandwidth. Stress control was applied during elastic deformation, switching to displacement control near the yield point to prevent creep‐induced strain artifacts under sustained loading. Neutron diffraction patterns were acquired for 1200 s per stress/strain increment to ensure statistical reliability. Rietveld refinement for multiple peak fitting of the neutron diffraction analysis was carried out using GSAS‐II [49].

### 5.7 Molecular Dynamics Simulations

MD simulations are performed using Large‐scale Atomic/Molecular Massively Parallel Simulator (LAMMPS) [50] with a general machine‐learned neuroevolution potential [51]. The simulation cell dimensions are 14.7 × 10 × 24.4 nm^3^, containing 314,400 atoms. The crystal orientations are [1$\bar{1}$2], [$\bar{1}$11], and [110] along x, y, z axes for the BCC phase, and [11$\bar{2}$], [1$\bar{1}$0], and [111] along x, y, z axes for the L1_2_ phase. Hence, the BCC/L1_2_ phase interface follows the Kurdyumov‐Sachs (K‐S) orientation relationship. Based on the sharp interface, an interface with Cr‐concentration gradient is introduced on the side of L1_2_ phase by randomly replacing Ni and Al atoms with Cr atoms. Specifically, the Cr concentration is 100% at the original sharp interface, and it decreases linearly along the normal direction of the interface and drops to 0% at a distance of ∼2 nm from the interface. Periodic boundary conditions are imposed in all three directions to avoid the boundary effect. A tensile loading is applied parallel to the interface along the x direction at 0 K with a small strain increment of 1 × 10^−6^, and the deformed system is subsequently relaxed under the conjugate gradient minimization. All the defective structures are identified by the common neighbor analysis and visualized by OVITO [52] during simulations.

### 5.8 Geometric Phase Analysis

The core principle of GPA lies in quantifying phase gradients relative to a reference lattice defined by a g‐vector. Here, distinct g‐vectors corresponding to crystallographic planes in L1_2_ and BCC phases were selected. GPA processing was performed using Strain++ software V1.8 [53].

## Author Contributions

P.G., B.Z., W.W., and Y.Y. supervised the project. P.G., Y.F., and Y.Y. conceived and designed the research. Z.L. performed the primary experiments. X.‐T.L. conducted simulations. Z.C. contributed to sample preparation and mechanical testing. Z.L., Y.G., W.X. performed TEM characterization. Z.C. and H.G. performed machine learning. H.G. and S.H. provided critical input on alloy design. W.S. executed in situ neutron diffraction tensile testing. C.L. and L.P. performed heat treatment experiments. Z.L., X.‐T.L., Y.F., P.G., and Y.Y. co‐wrote the manuscript. All authors contributed to data analysis and discussions and provided critical feedback on the manuscript.

## Conflicts of Interest

The authors declare no conflict of interest.

## Supporting information




**Supporting File**: advs74076‐sup‐0001‐SuppMat.docx.

## Data Availability

The data that support the findings of this study are available from the corresponding author upon reasonable request.
