# Supplementary material for: Transforming Grain‐Boundary Brittle Precipitates to Ductility Pathways in Complex Concentrated Alloy
Source: Adv Sci (Weinh). 2026 Feb 10;13(19):e18465. doi: 10.1002/advs.202518465 (PMC13045251; doi:10.1002/advs.202518465)
Supplement: Supplementary file 1 — Supporting File: advs74076‐sup‐0001‐SuppMat.docx. [file ADVS-13-e18465-s001.docx]

Supporting Information

Transforming Grain-Boundary Brittle Precipitates to Ductility Pathways in Complex Concentrated Alloy

Zhixin Li, Xiao-Tong Li, Zhaoqi Chen, Yushan Geng, Hao Gong, Sijia Hu, Wenli Song, Wanshun Xia, Chuanzheng Li, Linfa Peng, Yue Fan*, Pengfei Guan*, Bo Zhang*, Weihua Wang and Yong Yang*

Z. X. Li, Z. Q. Chen, Y. S. Geng, H. Gong, S. J. Hu, W. S. Xia, C. Z. Li, Y. Yang

Department of Mechanical Engineering, City University of Hong Kong, Tat Chee Avenue, Kowloon Tong, Kowloon, Hong Kong, 000997, China

E-mail: yonyang@cityu.edu.hk

Z. X. Li, B. Zhang, W. H. Wang

Songshan Lake Materials Laboratory, Dongguan, Guangdong 523808, China

E-mail: zhangbo@sslab.org.cn

X. -T. Li, P. F. Guan

Advanced Interdisciplinary Science Research (AiR) Center, Ningbo Institute of Materials Technology and Engineering, Chinese Academy of Sciences, Ningbo 315201, China

E-mail: pguan@nimte.ac.cn

X. -T. Li, P. F. Guan

State Key Laboratory of Advanced Marine Materials, Ningbo Institute of Materials Technology and Engineering, Chinese Academy of Sciences, Ningbo 315201, China

Y. S. Geng

State Key Laboratory of Solid Lubrication, Lanzhou Institute of Chemical Physics, Chinese Academy of Sciences, Lanzhou 730000, China

W. L. Song

Institute of High Energy Physics, Chinese Academy of Science (CAS), Beijing 100049, P.R. China

C. Z. Li, L. F. Peng

State Key Laboratory of Mechanical System and Vibration, Shanghai Jiao Tong University, Shanghai, 200240, P. R. China

Y. Fan

Department of Mechanical Engineering, University of Michigan, Ann Arbor, MI 48109, USA

E-mail: fanyue@umich.edu

W. L. Song

Spallation Neutron Source Science Center, Dongguan, Guangdong 523803, China

W. S. Xia

Institute of Superalloys Science and Technology, School of Materials Science and Engineering, Zhejiang University, Hangzhou 310027, Zhejiang, China

P. F. Guan

Beijing Computational Science Research Center, Beijing 100193, China

Y. Yang

Department of Materials Science and Engineering, City University of Hong Kong, Tat Chee Avenue, Kowloon Tong, Kowloon, Hong Kong, 000997, China

**This file includes:**

Supporting text

Figures S1 to S14

Tables S1 to S2

SI References

**Supporting Text**

**Segregation enthalpy calculations**

The effective grain boundary segregation enthalpy in ternary systems (A-B-C) is given by^[1]^:

$$\Delta H_{AC}^{seg-eff}=\Delta H_{AC}^{seg(bin)}+\frac{1}{3}(\Delta H_{AB}^{mix}-\Delta H_{AC}^{mix}-\Delta H_{BC}^{mix})$$

where $\Delta H_{AC}^{seg-eff}$is the effective segregation enthalpy of ternary system (A-B-C), $\Delta H_{AC}^{seg(bin)}$is the segregation enthalpy of binary system (A-C), $\Delta H_{AB}^{mix}$ is the mixing enthalpy of element A and B, extending to other elemental pairs. To implement this equation in the present alloy system, the following assumptions are introduced: (1) Ni constitutes up to 59 at.% and is therefore defined as the solvent. (2) Fe accounts for only 5 at.% with the similar atomic radius to Ni. Consequently, we ignore Fe’s negligible effect on elemental segregation, simplify this quaternary alloy to a pseudo-ternary Ni-Cr-Al configuration. All the binary mixing enthalpy was derived from OQMD database^[2]^. All binary segregation enthalpy was obtained from machine learning predicted segregation energy spectra^[3]^. Herein, we determine the elemental segregation tendencies at grain boundary by computing the grain boundary segregation enthalpy for Cr and Al in the present system. This approach yields the following calculations:

$$\Delta H_{NiCr}^{seg-eff}=\Delta H_{NiCr}^{seg\left( bin \right)}+\frac{1}{3}\left( \Delta H_{NiAl}^{mix}-\Delta H_{NiCr}^{mix}-\Delta H_{CrAl}^{mix} \right)=-3+\frac{1}{3}\times\left( -64.5487+1.2543+13.5079 \right)=-19.5956 kJ/mol$$

$$\Delta H_{NiAl}^{seg-eff}=\Delta H_{NiAl}^{seg(bin)}+\frac{1}{3}\left( \Delta H_{NiCr}^{mix}-\Delta H_{NiAl}^{mix}-\Delta H_{CrAl}^{mix} \right)=-2+\frac{1}{3}\times\left( -1.2543+64.5487+13.5079 \right)=23.6008 kJ/mol$$

**Strength-ductility index calculations**

Based on the conventional strength-ductility product^[4]^, we introduced a yield strength modification term, thus defining our strength-ductility index (SDI) formula as follows:

$$SDI= \frac{\sigma_{YS}}{1 \mathrm{GPa}}\times\sigma_{UTS}\times EL$$

Unit: $GPa\times\%$

where $\sigma_{YS}$ is yield stress, $\sigma_{UTS}$ is ultimate tensile stress, and $EL$ is fracture elongation.

**Neutron diffraction data analysis**

Lattice strain $\varepsilon_{hkl}$ was calculated from the TOF data by the following equation:

$$\varepsilon_{hkl}=\frac{{(d}_{hkl}-d_{hkl}^{0})}{d_{hkl}^{0}}=\frac{{(TOF}_{hkl}-{TOF}_{hkl}^{0})}{{TOF}_{hkl}^{0}}$$

where $d_{hkl}$ is the lattice spacing of $hkl$ planes, $d_{hkl}^{0}$ is the value for the stress-free sample, and ${TOF}_{hkl}$ is the Bragg position of the $hkl$ reflection in the TOF data.

Stacking faults probability (SFP) was calculated from the lattice strain by following equation^[5]^:

$$SFP=\frac{32\pi}{3\sqrt{3}}(\varepsilon_{222}-\varepsilon_{111})$$

where $\varepsilon_{222}$ and $\varepsilon_{111}$ are the experimentally measured lattice strain values with applied stress for (222) and (111) reflections, respectively.

**Energy dissipation calculations**

In each strain increment, the work done by the applied stress per unit volume is expressed as ${\Delta W}_{\sigma}=1/2(\sigma^{i}+\sigma^{i+1})\cdot\Delta\varepsilon$, and the total energy stored in the system is given by $\Delta U={\Delta W}_{\sigma}\cdot V$. A portion of this stored energy is converted into potential energy, denoted as ${\Delta P}_{e}$, which can be estimated using the difference: $P_{e}^{i+1}-P_{e}^{i}$. Consequently, the dissipated energy of the system is calculated as $\Delta E_{d}=\Delta U-{\Delta P}_{e}$. By collecting $\Delta E_{d}$ for samples with sharp and graded interfaces across the entire loading window, the dissipated energy spectra can be constructed. Figure 4G presents data for the scenario where $\Delta E_{d}\geq0.1$ eV after logarithmic transformation.

**Grain boundary strengthening calculation**

Grain boundary strengthening contribution was quantified using the Hall-Petch equation^[6]^:

$$\sigma_{y}=\sigma_{0}+k_{y}\cdot d^{-\frac{1}{2}}$$

where $\sigma_{y}$ is the yield stress, $\sigma_{0}$ is the friction stress, $k_{y}$ is the Hall-Petch coefficient and $d$ is the average grain size. $k_{y}$ is adopted as 710 MPa·μm¹/² from Inconel 718^[7]^, a widely used nickel-based alloy reference, due to the lack of material-specific coefficient for the novel nickel-based complex concentrated alloy.

For the 973 K-annealed sample (average grain size of ~0.4 μm), the calculated grain boundary strengthening value is ~1100 MPa. For the 1173 K-annealed sample (average grain size of ~0.8 μm), the calculated grain boundary strengthening value is ~800 MPa.

**Figures**

**Figure S1.** Schematic diagram of the ANN-based alloy design workflow.

**Figure S2.** Microstructure of homogenized-alloy (H-alloy). (A) Back scattered electron (BSE) image showing homogeneous matrix without precipitates or dendritic segregation. (B) Grain size distribution (>100 grains) quantified from (A). (C) Centered dark-field TEM (CDF-TEM) image revealing L1_2_ superlattice reflections. (C_1_-C_2_) Selective area electron diffraction (SAED) patterns of H alloy, viewed from zone axes [011] and [001], respectively. (D) High-resolution TEM (HR-TEM) image of the white square in (C). (D_1_) fast Fourier transformation (FFT) image of (D) confirming L1_2_ ordering. (D_2_) IFFT reconstruction derived from superlattice in (D_1_). (E_1_-E_4_) Scanning transmission electron microscopy-energy dispersive X-ray spectroscopy (STEM-EDS) mappings of Al, Cr, Fe and Ni, respectively. Arrows indicate Cr and Ni partitioning between FCC and L1_2_ phases.

**Figure S3.** Thermodynamic equilibrium phase fractions and elemental partitioning in Ni_59_Cr_24_Al_12_Fe_5_ (at%) from 700 to 2000 K. (A) Phase fractions. (B) Elemental concentrations in BCC phase. (C) Elemental concentrations in FCC phase. (D) Elemental concentrations in L1_2_ phase. Calculations performed with Thermo-calc 2024b.

**Figure S4.** Phase fraction of samples annealed at 973 K and 1173 K for 8 hours. (A) Phase fraction of 973 K-annealed sample, derived from EBSD. (B) Phase fraction of 1173 K-annealed sample, derived from EBSD. (C) Phase fraction of 973 K-annealed sample, derived from refined neutron diffraction. (D) Phase fraction of 1173 K-annealed sample, derived from refined neutron diffraction.

**Figure S5.** Chemical distribution at interface in alloys annealed at 973 K and 1173 K for 8 hours. (A_1_-A_4_) STEM-EDS mappings of Ni, Cr, Al and Fe at graded interface (GI) in 1173 K-annealed alloy, respectively. (B_1_-B_4_) STEM-EDS mappings of Ni, Cr, Al and Fe at sharp interface in 973 K-annealed alloy, respectively. (C_1_-C_2_) Line-scan compositional intensity profile of arrows in (A_1_ and B_1_), respectively. Scale bar = 2 nm.

**Figure S6** The results of tilt-angle TEM tests on samples annealed at 1173 K for 8 hours. (A) The STEM image includes L1_2_, GI and BCC; (A_1_ to A_4_) The corresponding STEM-EDS mappings of Ni, Cr, Al and Fe, respectively; (B) The edge-on high-resolution TEM images were taken in the white box area of (A) with L1_2_ zone axis (011). The inset is the FFT image of the white square region (C_1_ to C_4_) The high-resolution TEM images were taken in the white box area of (A) with other tilting angles.

**Figure S7.** Lattice strain evolution across BCC/L1_2_ graded interface (GI) in alloy annealed at 1173 K for 8 hours. (A) Aberration-corrected STEM-HAADF image of the GI between BCC and L_12_ phases. The inset is an FFT image from the white dashed area showing BCC and L1_2_ patterns in GI, viewed from the zone axis [111]BCC. (B_1_-B_3_) Lattice strain maps in (A) of ε_xx_, ε_xy_ and ε_yy_, respectively, derived from BCC spots 1 and 2. (C_1_-C_3_) Lattice strain maps in (A) of ε_xx_, ε_xy_ and ε_yy_, respectively, derived from L1_2_ spots 1 and 2.

**Figure S8.** Structural analysis in alloys annealed at 973 K and 1173 K for 8 hours. (A) Aberration-corrected STEM-HAADF image of the GI between BCC and L1_2_. (B) The corresponding FFT image of (A). (C) Aberration-corrected STEM-HAADF image of the sharp phase boundary between BCC and L1_2_. (D) The corresponding FFT image of (C). The spots indicated by yellow arrows were used to IFFT.

**Figure S9.** Post-fracture microstructural analysis of alloy annealed at 973 K for 8 hours. (A-C) Lattice strain evolution of FCC, BCC and L1_2_, respectively, upon in-situ neutron diffraction tension. (D) STEM-HAADF image showing dislocation-free BCC phase. (E-F) HR-TEM images of BCC and L1_2_ phases, respectively. The insets are corresponding FFT images. (G-H) IFFT images of BCC and L1_2_ phases, derived from the yellow circle in (E-H).

**Figure S10** Generalized stacking fault energy (GSFE) curves for Cr and Ni3Al. Defective structures correspond to the curve peaks or valleys are denoted: unstable stacking fault (USF) for Cr; unstable superlattice intrinsic stacking fault (USISF), superlattice intrinsic stacking fault (SISF), anti-phase boundary (APB), and complex stacking fault (CSF) for Ni3Al.

**Figure S11.** The Pole Figures of FCC phases in 973 K- and 1173 K-annealed samples for 8 hours. (A) The pole figures of FCC {100}, {110}, {111} planes in 973 K-annealed sample. (B) The pole figures of FCC {100}, {110}, {111} planes in 1173 K-annealed sample.

**Figure S12.** In-situ neutron diffraction analysis of tensile deformation mechanisms in alloys annealed at 973 K and 1173 K for 8 hours. (A) In-situ neutron diffraction patterns of 973 K-annealed alloy. The inset is the corresponding enlarged image revealing peak splitting in L1_2_ phase and elastic recovery in BCC phase. (B) In-situ neutron diffraction patterns of 1173 K-annealed alloy.

**Figure S13** Strain-stress curves and atomic snapshots for Cr and Ni_3_Al single crystals during uniaxial tensile loading. The black arrows indicate consistent slip directions of stacking faults in bulk Ni_3_Al.

**Figure S14.** Thermal stability and grain growth kinetics of 1173 K-annealed alloys. (A) Grain evolution identified by EBSD with increased annealing time at 1173 K. (B) Grain size versus annealing time compared with other alloys. (C) Enlarged plot of red dashed box in (B).

**Tables**

**Table S1.** Phase concentrations in alloys annealed at 973 K and 1173 K for 8 hours.

| Sample | Phase | Ni (at%) | Cr (at%) | Al (at%) | Fe (at%) |
| --- | --- | --- | --- | --- | --- |
| 973 K-annealed alloy | FCC | 63.55±4.10 | 26.40±3.58 | 4.33±0.46 | 5.72±0.92 |
|  | L1_2_ | 73.41±3.73 | 11.76±2.21 | 9.44±2.11 | 5.38±1.44 |
|  | BCC | 2.04±0.86 | 95.92±1.57 | 0.95±1.10 | 2.04±1.08 |
| 1173 K-annealed alloy | FCC | 61.52±4.14 | 26.35±3.53 | 4.87±0.54 | 7.27±1.15 |
|  | L1_2_ | 74.62±2.99 | 9.86±1.61 | 11.52±1.30 | 4.00±0.69 |
|  | BCC | 2.57±0.51 | 94.76±0.85 | 0.65±0.23 | 2.02±0.39 |

**Table S2.** Composition, processing, phase, tensile properties, and raw materials cost of alloys studied previously.

| Type | Alloys | Processing | Phase | σ_YS_ (MPa) | σ_UTS_ (MPa) | ε (%) | RMC(USD/kg) | SDI(GPa×%) | Ref. |
| --- | --- | --- | --- | --- | --- | --- | --- | --- | --- |
| TW | Ni_59_Cr_24_Al_12_Fe_5_ | H | FCC + L1_2_ | 435.48 | 685.80 | 37.85 | 11.39719 | 11.30399 |  |
|  |  | H + CR80% + 1173 K 30 min | FCC + L1_2_ + BCC | 1181.96 | 1399.85 | 19.29 |  | 31.91659 |  |
|  |  | H + CR80% + 1173 K 2 h | FCC + L1_2_ + BCC | 1000.47 | 1260.00 | 21.10 |  | 26.5985 |  |
|  |  | H + CR80% + 1173 K 8 h | FCC + L1_2_ + BCC | 912.08 | 1170.86 | 26.22 |  | 28.00081 |  |
|  |  | H + CR80% + 1173 K 24 h | FCC + L1_2_ + BCC | 849.37 | 1097.47 | 28.22 |  | 26.3055 |  |
|  |  | H + CR80% + 1173 K 48 h | FCC + L1_2_ + BCC | 808.26 | 1088.75 | 34.37 |  | 30.24536 |  |
|  |  | H + CR80% + 1173 K 80 h | FCC + L1_2_ + BCC | 712.81 | 1020.98 | 36.10 |  | 26.27231 |  |
|  |  | H + CR80% + 1173 K 120 h | FCC + L1_2_ + BCC | 686.09 | 1000.63 | 36.95 |  | 25.367 |  |
|  |  | H + CR80% + 973 K 30 min | FCC + L1_2_ + BCC | 1519.13 | 1519.13 | 0.74 |  | 1.707739 |  |
|  |  | H + CR80% + 973 K 2 h | FCC + L1_2_ + BCC | 1942.67 | 1942.67 | 1.07 |  | 4.038144 |  |
|  |  | H + CR80% + 973 K 8 h | FCC + L1_2_ + BCC | 1586.53 | 1584.13 | 1.27 |  | 3.191853 |  |
|  |  | H + CR80% + 973 K 24 h | FCC + L1_2_ + BCC | 1374.28 | 1375.95 | 1.43 |  | 2.704045 |  |
|  |  | H + CR80% + 973 K 48 h | FCC + L1_2_ + BCC | 1348.21 | 1348.21 | 3.04 |  | 5.525717 |  |
|  |  | H + CR80% + 973 K 120 h | FCC + L1_2_ + BCC | 1260.23 | 1283.72 | 6.15 |  | 9.949362 |  |
| SP | Al_6_Cr_25_Fe_34_Ni_35_ | CR + Recrystallized | FCC | 456 | 685 | 22 | 7.732184 | 6.87192 | ^[8]^ |
| SP | CoCrMnNi | AC + 1373 K 24 h + CR90% + 1273 K 1 h | FCC | 280 | 699 | 43 | 14.86277 | 8.41596 | ^[9]^ |
| SP | CoFeMnNi | AC + 1373 K 24 h + CR90% + 1273 K 1 h | FCC | 175 | 551 | 41 | 12.57613 | 3.953425 |  |
| SP | Cr_0.66_FeMnNi | AC + 1473 K 24 h + CR 86% + 1173 K 1 h | FCC | 265 | 630 | 37 | 6.192946 | 6.17715 | ^[10]^ |
| SP | HfTaTiZr | AC | BCC | 1356 | 1452 | 4 | 443.3864 | 7.875648 | ^[11]^ |
| SP | HfTa_0.6_TiZr | AC | BCC | 750 | 1110 | 22.1 | 465.7018 | 18.39825 |  |
| SP | Fe_40_Mn_40_Ni_10_Cr_10_ | AC | FCC | 293 | 460 | 53.8 | 3.248806 | 7.251164 | ^[12]^ |
| SP | Fe_40_Ni_20_Co_20_Cr_20_ | HR + H + CR + Annealed | FCC | 559 | 612 | 29 | 11.67726 | 9.921132 | ^[13]^ |
| SP | Al_0.5_CoCrCuFeNi | AC | FCC | 360 | 707 | 19.1 | 12.12116 | 4.861332 | ^[14]^ |
| SP | Al_8_Co_17_Cr_17_Cu_8_Fe_17_Ni_33_ | AC | FCC | 357 | 459 | 9 | 13.08739 | 1.474767 | ^[15]^ |
| SP |  | 973 K 5 h | FCC | 365 | 365 | 0.1 |  | 0.013323 |  |
| SP |  | 1423 K 5 h | FCC | 215 | 489 | 0.02 |  | 0.002103 |  |
| SP | Co_20_Cr_40_Ni_40_ | AC | FCC | 373 | 680 | 69 | 16.20412 | 17.50116 | ^[16]^ |
| SP | Co_40_Cr_30_Ni_30_ | AC | FCC | 364 | 750 | 53 | 20.51042 | 14.469 |  |
| SP | Co_60_Cr_20_Ni_20_ | AC | FCC | 441 | 666 | 16 | 24.70921 | 4.699296 |  |
| SP | CoCrCuFeNi | AC | FCC | 230 | 888 | 50.76 | 12.60342 | 10.36722 | ^[17]^ |
| SP | CoCrFeNi | AC | FCC | 188 | 457 | 50 | 14.46454 | 4.2958 | ^[18]^ |
| SP |  | AC + 1273 K 24 h + CR80% + 898 K 1 h | FCC | 540 | 786 | 49.3 |  | 20.92489 |  |
| SP |  | AC + 1273 K 24 h + 1273 K + HR92% + 1173 K 1 h | FCC | 300 | 671 | 42 |  | 8.4546 | ^[19]^ |
| SP |  | AC + 1473 K 24 h + 1273 K + CR92% + 1173 K 1 h | FCC | 273 | 714 | 38 |  | 7.407036 | ^[9]^ |
| SP |  | SLM 50 μm per layer | FCC | 402 | 480 | 8 |  | 1.54368 | ^[18]^ |
| SP |  | SLM 20 μm per layer | FCC | 600 | 745 | 32 |  | 14.304 |  |
| SP |  | SLM 20 μm per layer + 1023 K 12 h | FCC | 495 | 695 | 30 |  | 10.32075 |  |
| SP |  | SLM 20 μm per layer + 1273 K 12 h | FCC | 433 | 682 | 42 |  | 12.40285 |  |
| SP | HfNbTaTiZr | AC | BCC | 805 | 888 | 9.4 | 387.1834 | 6.719496 | ^[20]^ |
| SP |  | AC + HIP 1473 K 207 MPa 2h + 1473 K 24 h + CR 86.4 % | BCC | 1202 | 1295 | 4.7 |  | 7.315973 | ^[21]^ |
| SP |  | AC + HIP 1473 K 207 MPa 2h + 1473 K 24 h + CR 86.4 % + 1073 K 2 h | BCC | 1303 | 1334 | 1.9 |  | 3.302584 |  |
| SP |  | AC + HIP 1473 K 207 MPa 2h + 1473 K 24 h + CR 86.4 % + 1273 K 2 h | BCC | 1145 | 1262 | 9.7 |  | 14.0164 |  |
| SP |  | H + CR + Recrystallized | BCC | 875 | 888 | 22.8 |  | 17.7156 | ^[22]^ |
| SP | Hf_0.5_Nb_0.5_Ta_0.5_Ti_1.5_Zr | AC | BCC | 903 | 990 | 18.8 | 299.9751 | 16.80664 | ^[23]^ |
| SP | ZrV_0.5_Nb_0.5_Ti | AC | BCC | 832 | 913 | 18.6 | 84.00903 | 14.12886 | ^[24]^ |
| SP | Nb_5_Ti_45_Zr_45_Mo_5_ | AC | BCC | 821 | 849 | 9.1 | 32.83214 | 6.342964 | ^[25]^ |
| SP | Nb_5_Ti_42.5_Zr_42.5_Ta_5_Mo_5_ | AC | BCC | 907 | 953 | 14.9 | 65.57951 | 12.87913 |  |
| DP | Al_0.7_Fe_2_Mn_1.8_Ni | AC | FCC+B2 | 270 | 580 | 23 | 3.737434 | 3.6018 | ^[26]^ |
| DP |  | AC + 1000 K 1 h | FCC + B2 | 420 | 780 | 22 |  | 7.2072 |  |
| DP |  | AC + 1000 K 1 h | FCC + B2 | 483 | 880 | 15 |  | 6.3756 |  |
| DP | HfTa_0.5_TiZr | AC | BCC + HCP | 687 | 1119 | 29.9 | 472.5176 | 22.98571 | ^[11]^ |
| DP | HfTa_0.4_TiZr | AC | BCC + HCP | 345 | 1126 | 30.6 | 479.966 | 11.88718 |  |
| DP | Fe_50_Mn_30_Co_10_Cr_10_ | H + CR + Recrystallized | BCC + HCP | 345 | 855 | 72 | 5.117624 | 21.2382 | ^[27]^ |
| DP | Fe_30_Ni_20_Mn_35_Al_15_ | AC | FCC + B2 | 810 | 1060 | 6.5 | 4.105073 | 5.5809 | ^[28]^ |
| DP | AlCoCrFeNi_2.1_ | CR90% + 1473 K 1 h | FCC + B2 | 648 | 1075 | 27 | 13.27079 | 18.8082 | ^[29]^ |
| DP |  | AC | L1_2_ + B2 | 620 | 1050 | 17 |  | 11.067 |  |
| DP |  | CR90% + 1073 K | FCC + B2 | 1108 | 1200 | 12 |  | 15.9552 |  |
| DP |  | CR90% + 1273 K 1 h | FCC + B2 | 844 | 1175 | 23 |  | 22.8091 |  |
| DP | Co_30_Cr_10_Fe_10_Al_18_Ni_30_Mo_2_ | AC | FCC + BCC | 750 | 1434 | 13.71 | 18.19456 | 14.74511 | ^[30]^ |
| DP | AlCrFeNi_3_ | AC | FCC + B2 | 626 | 1200 | 10.1 | 9.675835 | 7.58712 | ^[31]^ |
| DP | (TiZrNbTa)_98.5_Al_1.5_ | AC | BCC1 + BCC2 | 11263 | 1150 | 20.7 | 1.64.8772 | 26.73302 | ^[32]^ |
| DP | Ti_80_Cr_6_Mo_5_V_5_Al_4_ (wt.%) | 1073 K 30 min | BCC1 + BCC2 | 798 | 798 | 18 | 31.22432 | 11.46247 | ^[33]^ |
| DP | Ni_72.7_Cr_14.1_Al_6_Mo_4.1_Nb_2_Ti_0.8_Fe_0.3_(wt.%) | AC | FCC + L1_2_ | 722 | 769 | 6.4 | 15.032888 | 3.553395 | ^[34]^ |
| DP | Ni_58.4_Cr_14.2_Al_4_Ti_2.6_Co_10.1_W_4.2_Mo_1.6_Ta_4.9_(wt.%) | 1393 K 2 h + 1123 K 24 h | FCC + L1_2_ | 988 | 1081 | 5.5 | 30.67884 | 5.874154 | ^[35]^ |
| DP | Ni_60_Cr_19.8_W_18.5_Mo_1.2_Al_0.5_(wt.%) | HR + 1533 K 0.5 h | FCC + L1_2_ | 439 | 873 | 49.9 | 17.24029 | 19.12403 | ^[36]^ |
| DP | K213 | 1373 K 4 h | FCC + L1_2_ | 745 | 990 | 4 | 8.927336 | 2.9502 | ^[37]^ |
| DP | K409 | AC | FCC + L1_2_ | 900 | 1060 | 12 | 29.78316 | 11.448 |  |
| MP | FeNi_0.9_Cr_0.5_Al_0.4_ | AC | FCC + BCC + B2 | 670 | 1196 | 21.1 | 7.023453 | 16.90785 | ^[38]^ |
| MP | AlCrFe_2_Ni_2_ | AC | FCC + BCC + B2 | 633 | 1092 | 14 | 7.193811 | 9.677304 | ^[39]^ |
| MP |  | As-printed | FCC + BCC + B2 | 960 | 1230 | 10.3 |  | 12.16224 | ^[40]^ |
| MP |  | As-printed + CR + 1073 K 1 h | FCC + BCC + B2 | 881 | 1174 | 10 |  | 10.34294 |  |
| MP |  | As-printed + CR + 1273 K 1 h | FCC + BCC + B2 | 758 | 1084 | 20.6 |  | 16.92644 |  |
| MP |  | As-printed + CR + 1473 K 1 h | FCC + BCC + B2 | 461 | 988 | 24 |  | 10.93123 |  |
| MP | AlCoCrFeNi_2.1_ | Directional solidification (30 μm/s) | FCC(L1_2_) + BCC(B2) | 397 | 1023 | 37.8 | 13.27079 | 15.35175 | ^[41]^ |
| MP |  | Directional solidification (60 μm/s) | FCC(L1_2_) + BCC(B2) | 418 | 1139 | 37.3 |  | 17.7586 |  |
| MP |  | Directional solidification (120 μm/s) | FCC(L1_2_) + BCC(B2) | 455 | 960 | 32.5 |  | 14.196 |  |
| MP | Al_0.8_CrFeNi_2.2_ | AC | FCC + BCC + B2 | 479 | 956 | 12.7 | 9.073293 | 5.815635 | ^[42]^ |
| MP | Cu_1.2_NiMnFe_0.5_Cr_0.5_Al_0.2_ | CR60% + 1323 K 2 h + 673 K 6 h | FCC1 + FCC2 + BCC | 701 | 1050 | 21.8 | 6.593013 | 16.04589 | ^[43]^ |
| MP | K4169(Inconel 718C) | 1368 K 1 h + Air cooling to 1228 K 1h + Air cooling to 993 K 8 h + Furnace cooling to 893 K 8 h + Air cooling | FCC + L1_2_ + BCT | 935 | 1100 | 16 | 22.52468 | 16.456 | ^[37]^ |
| MP | GH4169(Inconel 718) | 1368 K 1 h + Air cooling to 1228 K 1h + Air cooling to 993 K 8 h + Furnace cooling to 893 K 8 h + Air cooling | FCC + L1_2_ + BCT | 1030 | 1280 | 12 | 11.49306 | 15.8208 |  |

Denotes: The following acronyms are used: σ_YS_ (yield strength); σ_UTS_ (ultimate tensile strength); ε (fracture elongation); RMC (raw materials cost); SDI (strength-ductility index); H (homogenized); AC (as-cast); CR (cold-rolled); HR (hot-rolled); SLM (selective laser melting); HIP (Hot isostatic pressing); TW (this work); SP (single-phase); DP (dual-phase); MP (multi-phase). RMC was calculated according to data from https://en.wikipedia.org/wiki/Prices_of_chemical_elements.

**References**

[1]	W. Xing, A. R. Kalidindi, D. Amram, C. A. Schuh, *Acta Materialia* **2018**, *161*, 285.

[2]	S. Kirklin, J. E. Saal, B. Meredig, A. Thompson, J. W. Doak, M. Aykol, S. Rühl, C. Wolverton, *npj Comput Mater* **2015**, *1*, 1.

[3]	M. Wagih, P. M. Larsen, C. A. Schuh, *Nat Commun* **2020**, *11*, 6376.

[4]	T. Yang, Y. L. Zhao, Y. Tong, Z. B. Jiao, J. Wei, J. X. Cai, X. D. Han, D. Chen, A. Hu, J. J. Kai, K. Lu, Y. Liu, C. T. Liu, *Science* **2018**, *362*, 933.

[5]	Muhammad Naeem, H. He, F. Zhang, H. Huang, S. Harjo, T. Kawasaki, B. Wang, S. Lan, Z. Wu, F. Wang, Y. Wu, Z. Lu, Z. Zhang, C. T. Liu, X.-L. Wang, *Science Advances* **2020**, *6*, eaax4002.

[6]	N. Hansen, *Scripta Materialia* **2004**, *51*, 801.

[7]	S. Zhang, X. Lin, L. Wang, X. Yu, Y. Hu, H. Yang, L. Lei, W. Huang, *Materials Science and Engineering: A* **2021**, *812*, 141145.

[8]	K. Lu, H. Shi, A. Weisenburger, J. Aktaa, *Materials Characterization* **2024**, *207*, 113578.

[9]	Z. Wu, H. Bei, G. M. Pharr, E. P. George, *Acta Materialia* **2014**, *81*, 428.

[10]	Z. Wu, H. Bei, *Materials Science and Engineering: A* **2015**, *640*, 217.

[11]	H. Huang, Y. Wu, J. He, H. Wang, X. Liu, K. An, W. Wu, Z. Lu, *Advanced Materials* **2017**, *29*, 1701678.

[12]	L. Bai, Y. Liu, Y. Guo, Y. Lv, T. Guo, J. Chen, *Journal of Alloys and Compounds* **2021**, *879*, 160342.

[13]	J. Chen, Z. Fang, P. Wu, H. Liu, L. Hu, T. Tuoliken, Z. Li, Q. Sun, *Materials Science and Engineering: A* **2024**, *909*, 146816.

[14]	F. Wang, Y. Zhang, G. Chen, H. A. Davies, *Int. J. Mod. Phys. B* **2009**, *23*, 1254.

[15]	H. M. Daoud, A. Manzoni, R. Völkl, N. Wanderka, U. Glatzel, *JOM* **2013**, *65*, 1805.

[16]	S. Yoshida, T. Ikeuchi, Y. Bai, N. Tsuji, *Materials Transactions* **2020**, *61*, 587.

[17]	X. Wang, Y. Zhang, Y. Qiao, G. Chen, *Intermetallics* **2007**, *15*, 357.

[18]	Y. Brif, M. Thomas, I. Todd, *Scripta Materialia* **2015**, *99*, 93.

[19]	G. A., E. P. George, *Intermetallics* **2013**, *39*, 74.

[20]	G. F. Dirras, L. Lola, P. Djémia, L.-B. Mathilde, D. Tingaud, J. P. Couzinié, L. Perrière, T. Chauveau, I. Guillot, *Materials Science and Engineering: A* **2016**, *654*, 30.

[21]	O. N. Senkov, S. L. Semiatin, *Journal of Alloys and Compounds* **2015**, *649*, 1110.

[22]	Shubin Wang, M. Wu, D. Shu, G. Zhu, D. Wang, B. Sun, *Acta Materialia* **2020**, *201*, 517.

[23]	S. Sheikh, S. Shafeie, Q. Hu, J. Ahlström, C. Persson, J. Veselý, J. Zýka, U. Klement, S. Guo, *Journal of Applied Physics* **2016**, *120*, 164902.

[24]	Y. Chen, Z. Xu, M. Wang, Yunkai Li, C. Wu, Y. Yang, *Materials Science and Engineering: A* **2020**, *792*, 139774.

[25]	W. Lai, H. Liu, X. Yu, Y. Yi, W. Li, S. Zhou, S. Cui, X. Wang, *Materials Science and Engineering: A* **2021**, *813*, 141135.

[26]	Z. Wang, I. Baker, *Materials Science and Engineering: A* **2017**, *693*, 101.

[27]	Z. Li, K. G. Pradeep, Y. Deng, D. Raabe, C. C. Tasan, *Nature* **2016**, *534*, 227.

[28]	F. Meng, J. Qiu, I. Baker, *Materials Science and Engineering: A* **2013**, *586*, 45.

[29]	W. I. Samad, B. Tilak, S. Saad Ahmed, P. P. Bhattacharjee, S. Guo, N. Tsuji, *Materials Science and Engineering: A* **2016**, *675*, 99.

[30]	Z. Yang, Z. Wang, Q. Wu, T. Zheng, P. Zhao, J. Zhao, J. Chen, *Appl. Phys. A* **2019**, *125*, 208.

[31]	Y. Dong, Z. Yao, X. Huang, F. Du, C. Li, A. Chen, F. Wu, Y. Cheng, Z. Zhang, *Journal of Alloys and Compounds* **2020**, *823*, 153886.

[32]	Y. Chen, Y. Fang, R. Wang, Y. Tang, S. Bai, Q. Yu, *Journal of Materials Science & Technology* **2023**, *141*, 149.

[33]	W. Chen, G. Yu, K. Li, Y. Wang, J. Zhang, J. Sun, *Materials Science and Engineering: A* **2021**, *811*, 141052.

[34]	M. F. Moreira, L. B. Fantin, F. Beneduce Neto, C. R. F. Azevedo, *Inter Metalcast* **2021**, *15*, 1129.

[35]	G. Yang, Y. Xu, L. Jiang, S. Liang, *Progress in Natural Science: Materials International* **2011**, *21*, 418.

[36]	G. Bai, J. Li, R. Hu, Z. Tang, X. Xue, H. Fu, *Materials Science and Engineering: A* **2011**, *528*, 1974.

[37]	Editorial Committee, *China Aeronautical Materials Handbook*, Vol. 2 Superalloys and Cast Superalloys, Standards Press of China **2001**.

[38]	Z. Jiang, W. Chen, C. Chu, Z. Fu, J. Ivanisenko, H. Wang, S. Peng, Y. Lu, E. J. Lavernia, H. Hahn, *Scripta Materialia* **2023**, *230*, 115421.

[39]	T. Bikash, P. P. Bhattacharjee, M. S. R.K., *Materials Science and Engineering: A* **2022**, *831*, 142190.

[40]	S. Mooraj, X. Dong, S. Zhang, Y. Zhang, J. Ren, S. Guan, C. Li, R. Naorem, N. Argibay, W. Chen, W. Yan, D. Raabe, Z. Sun, W. Chen, *Commun Mater* **2024**, *5*, 1.

[41]	L. Wang, C. Yao, J. Shen, Y. Zhang, T. Wang, Y. Ge, L. Gao, G. Zhang, *Intermetallics* **2020**, *118*, 106681.

[42]	Xi Jin, Juan Bi, Lu Zhang, Yang Zhou, Xingyu Du, Yuxin Liang, Bangsheng Li, *Journal of Alloys and Compounds* **2019**, *770*, 655.

[43]	Y. Wu, D. Zhao, B. Zheng, X. Liu, X. Hui, *Journal of Materials Research and Technology* **2024**, *30*, 7958.
